# Fecal Microbiota Transplantation Donor and Dietary Fiber Intervention Collectively Contribute to Gut Health in a Mouse Model

**DOI:** 10.3389/fimmu.2022.842669

**Published:** 2022-02-03

**Authors:** Yifan Zhong, Jiahong Cao, Yanfei Ma, Yu Zhang, Jianxin Liu, Haifeng Wang

**Affiliations:** College of Animal Science, Zhejiang University, The Key Laboratory of Molecular Animal Nutrition, Ministry of Education, Hangzhou, China

**Keywords:** fecal microbiota transplantation, dietary fiber, gut microbiota, metabolomics, gut barrier

## Abstract

Transforming the gut microbiota has turned into the most intriguing target for interventions in multiple gastrointestinal and non-gastrointestinal disorders. Fecal microbiota transplantation (FMT) is a therapeutic tool that administers feces collected from healthy donors into patients to help replenish the gut microbial balance. Considering the random donor selection, to maintain the optimal microbial ecosystem, post-FMT is critical for therapy outcomes but challenging. Aiming to study the interventions of different diets on recipients’ gut microbiota post-FMT that originated from donors with different diets, we performed FMT from domestic vs. wild pigs that are living on low-fiber vs. high-fiber diets into the pseudo-GF mouse, followed with fiber-free (FF) or fiber-rich (FR) diets post-FMT. Different patterns of gut microbiota and metabolites were observed when mice FMT from different donors were paired with different dietary fiber contents. Enrichment of bacteria, including *Akkermansia* and *Parabacteroides*, together with alteration of metabolites, including palmitic acid, stearic acid, and nicotinic acid, was noted to improve crypt length and mucus layer in the gut in mice FMT from wild pigs fed an FR diet. The results provide novel insight into the different responses of reconstructed gut microbiota by FMT to dietary fiber. Our study highlighted the importance of post-FMT precise dietary interventions.

## Introduction

Dietary fiber plays a vital role in gut health and shapes gut microbiota by serving as an important substrate and a major energy source for gut microbiota (1, 2). Gut microbiota together with their metabolic compounds could contribute to the gut health of the host or pathogenesis of diseases (3). Insoluble forms of dietary fiber such as cellulose can increase the transit rate of non-digested foodstuff and be fermented by bacteria in the colon. Soluble forms of dietary fiber such as inulin can be fermented by gut microbiota and stimulate the production of metabolites, which are beneficial to the physiology of the host (4). Numerous studies have demonstrated the beneficial aspects of high dietary fiber, while low dietary fiber intake usually contributes to a disruption of gut microbiota and metabolism in the gut (5, 6). The composition and function of gut microbiota were altered when different levels of dietary fiber were introduced. The higher abundances of *Lactococcus*, *Eggerthella*, and *Streptococcus* were found in the gut together with lower levels of short-chain fatty acids (SCFAs) when humans are fed a low-fiber diet (7). As dietary fiber is implemented and digested by gut microbiota, specific metabolites such as SCFAs can be produced to maintain gut barrier integrity (8, 9). The western diet contains low dietary fiber and is suggested to have irreversibly damaged gut microbiota with the disappearance of many bacterial species (10, 11). Mice transplanted with human bacteria and a diet intake lacking fiber have been shown to have reduced microbial diversity in the gut within three generations. What is more, gut microbiota were difficult to restore when a normal fiber diet was then introduced (12).

As a therapeutic approach, fecal microbiota transplantation (FMT) has been applied in the restoration of a disturbed microbial ecosystem and metabolism in the gut, leading to the attenuation of inflammatory or disorder in the recipient (13). Despite that FMT has been demonstrated as an effective practice especially in the treatment of inflammatory bowel disease (IBD) (14, 15), contradictory results of the FMT trials were still observed (16). In the process of FMT, the selection of donors was the first but critical point since gut microbiota composition could vary within healthy individuals (17), which interact between the donor and recipient microbiota at both taxonomical and functional levels (13). Moreover, although patterns of gut microbiota of humans with differential dietary intake were demonstrated (18, 19), the effect of dietary intake of both the donor and recipient during the FMT process has still begun to be further explored (20, 21).

Pigs are one of the earliest domesticated livestock species whose ancestors still exist in large numbers in the wild (22). Similarities in the functional pathways of human and pig gut microbiota provided evidence for an ideal model for human research (23). Unlike domestic pigs that received an “industrialized” diet with low dietary fiber contents, wild pigs mainly feed on a “non-industrialized” diet that includes acorns, wild fruits, grassroots, and stems with high cellulose content and low carbohydrate or fat content. Thus, domestic pigs and wild pigs were selected as models for donors with different dietary fiber intakes.

In this study, pseudo-germ-free mice models were established, and FMT was performed on domestic and wild pigs subsequently, further manipulated with differential dietary fiber contents. We aim to investigate the dynamics of gut microbiota and metabolites in recipient FMT from donors with differential dietary fiber contents, which will provide new insight into the influence of dietary fiber intake on FMT and gut health.

## Materials and Methods

### Animals and Design

**Figure 1:**
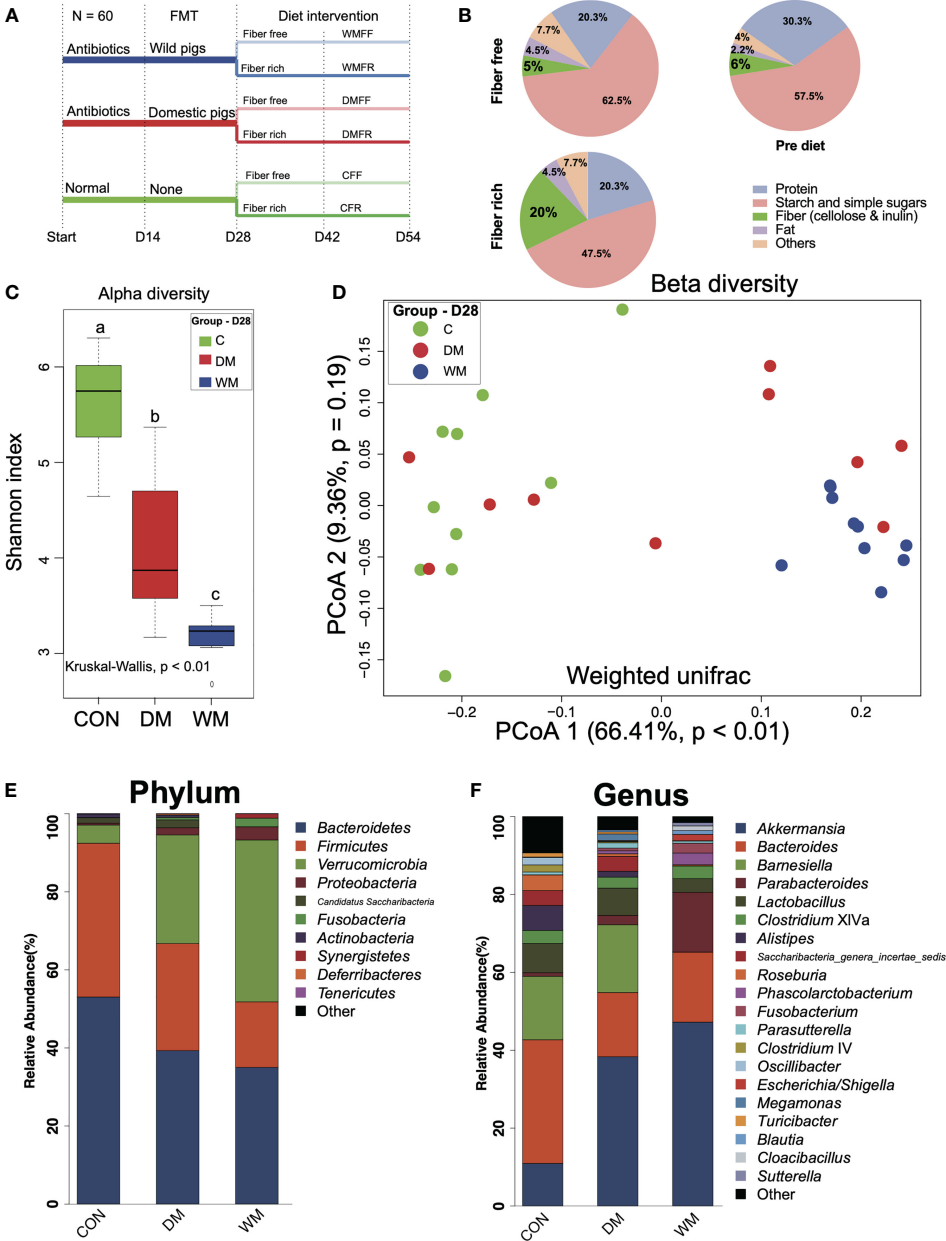
Timeline of trial schematic, diet composition, and bacteria patterns of mice after fecal microbiota transplantation (FMT). **(A)** Schematic of the mouse model illustrating the timeline of antibiotic treatment, fecal microbiota transplantation (FMT), feeding strategies, and fecal sampling. **(B)** Composition of the diets employed in this study. **(C)** Shannon index of the control group (CON), mice FMT with domestic pigs (DM), and wild pigs (WM). **(D)** Principal coordinates analysis (PCoA) of gut microbiota structures in the mice of the CON, DM, and WM groups. **(E, F)** Bar plot of phylum and genus levels in the mice of CON, DM, and WM groups. Kruskal–Wallis test with an adjusted *p-*value of <0.05 was applied. ^a,b,c^ Means within the same row followed by different superscripts differ at *p* < 0.05.

All procedures involving animals were performed in full accordance with and approved by the Animal Care and Use Committee of Zhejiang University (ethics code permit no.: ZJU20170529). Sixty male Institute of Cancer Research (ICR) mice (weight: 20 ± 2 g; age, 8 weeks) were obtained from the Model Animal Research Center of Nanjing University (Nanjing, China). Mice were maintained at 25°C in a 12-h light–dark cycle and had *ad libitum* access to food and water. The study procedure is shown in **Figure 1A**. In brief, forty mice were treated with antibiotics for 14 days and FMT from domestic pig microbiota (DM) or wild pig microbiota (WM), while the rest of the mice as control (CON) were free from antibiotics and FMT. After FMT (day 28), each group was further divided into two subgroups receiving a fiber-free (FF) or fiber-rich (FR) diet. The composition of pre-diet, FF diet, and FR diet applied in the study is shown in **Figure 1B** and **Table S1**.

### Fecal Microbiota Transplantation

Mice were treated with a cocktail of four antibiotics (ampicillin, 1 g/L; vancomycin, 500 mg/L; neomycin sulfate, 1 g/L; and metronidazole, 1 g/L) for 14 days. Fresh feces were collected from adult wild pigs (Zhejiang Province, China) and domestic pigs (Duroc × Landrace × Large White) and immediately diluted with sterile phosphate-buffered saline (PBS) (1 g/10 ml) and centrifuged at 1,000 rpm at 4°C for 5 min. The suspension was mixed with an equal volume of 40% sterile glycerol to a final concentration of 20% and then stored at −80°C. For FMT, the DM and WM mice were dosed by means of oral gavage with 200 μl of bacterial suspension from domestic pigs or wild pigs every other day for 14 days. CON mice were given 200 μl of PBS solution containing 20% glycerol instead.

### Collection of Mouse Fecal and Colonic Samples

Fresh fecal pellets were collected on day 14 (after antibiotic treatment), day 28 (after FMT), and days 42 and 54 (after dietary fiber intervention) and stored at −80°C. At the endpoint, mice were euthanized *via* an intravenous injection of sodium pentobarbital (50 mg/kg body weight). Segments of the colon were gently flushed twice with 5 ml of 0.9% saline for use in histopathological and mucus-layer analyses.

### H&E and Immunofluorescence Staining

H&E staining was performed as previously described (4). Briefly, colonic tissue samples were soaked, covered, and sliced into sections. Sections were soaked and subsequently stained with H&E. Photomicrographs were obtained *via* optical microscopy, and colonic crypt length was determined using Imaging Software (CS-EN-V1.18) (Olympus Corporation, Tokyo, Japan). The thickness of the colonic inner mucus layer was measured from the Alcian blue-stained slides and validated *via* anti-MUC2 staining. Immunofluorescence staining for MUC2 was performed as previously described (24, 25). Briefly, colon samples were stained with a 1:200 dilution of MUC2 primary antibody (Biorbyt, LLC, San Francisco, CA, USA) and anti-rabbit Alexa Fluor488 secondary antibody diluted 1:500 (Thermo Fisher Scientific, Waltham, MA, USA). The slides were then visualized with an Olympus BX63 upright fluorescence microscope, and mucus layer thickness was measured by using Imaging Software (V1.18) (Olympus, Tokyo, Japan).

### Serum Parameters

Serum samples were collected from the hearts of the mice at the endpoint. Concentrations of total cholesterol (TC), high-density lipoprotein cholesterol (HDL-C), low-density lipoprotein cholesterol (LDL-C), triglycerides (TG), glucose, and d-xylose as well as insulin activity in the serum were determined using commercial kits (Nanjing Jiancheng Bioengineering Institution, Nanjing, China) in an automatic biochemistry analyzer (SELECTA XL; Vital Scientific, Newton, MA, USA) according to the manufacturer’s instructions.

### Short-Chain Fatty Acid Analysis

Fecal samples were homogenized and centrifuged at 10,000 × *g* for 10 min, and the supernatant was filtered through a 0.22-μm filter for quantitation of SCFAs. High-performance liquid chromatography (HPLC) was performed using an Agilent 6890N gas chromatography system (Agilent, CA, USA) according to the manufacturer’s instructions.

### 16S rRNA Sequencing

The V3–V4 region of the bacterial 16S rRNA gene was amplified using primers 341F (5′-CCTACGGGRSGCAGCAG-3′) and 806R (5′-GGACTACVVGGGTATCTAATC-3′) (26). PCRs were performed, and the library was constructed and assessed using Qubit. Finally, the library was sequenced on an Illumina HiSeq PE2500 platform, and 250-bp paired-end reads were generated (27). Sequences were achieved, and chimeric reads were removed by Userach (V7.0.1090). Operational taxonomic units (OTUs) were selected *via* standard clustering with 97% similarity using UPARSE. Each representative tag was assigned to a taxon using the RDP Classifier (http://rdp.cme.msu.edu). OTU abundance tables were obtained, and QIIME1 (v1.9.1) was implemented for OTU profiling, alpha/beta diversity, and rank abundance curve analyses. Linear discriminant analysis (LDA) effect size (LEfSe) and rank sum test (R version 3.5.1) were used to screen differential bacteria within six groups.

### Availability of Sequencing Data

The DNA sequences of this article were deposited in the National Center for Biotechnology Information (NCBI) Sequence Read Archive (SRA) repository under accession number PRJNA723114.

### Metabolomic Analysis

Fecal samples were centrifuged, and supernatant (0.28 ml) was obtained and (28) analyzed by gas chromatography/time-of-flight mass spectrometry (GC-TOF-MS) using a 7890 Gas Chromatograph System (Agilent Technologies, Santa Clara, CA, USA) coupled with a Pegasus™ HT TOF Mass Spectrometer (LECO, Saint Joseph, MI, USA). Chroma TOF 4.3x software (LECO) and LECO-Fiehn Rtx5 database were used for extracting raw peaks, filtering, calibrating baselines, aligning peaks, performing deconvolution analysis, identifying peaks, and integrating peak area (29). Retention time index (RI) was used for peak identification, with an RI tolerance of 5,000. Metabolic features detected in <50% of quality control (QC) samples were removed (30). The identified differential metabolites were further validated by searching in the Kyoto Encyclopedia of Genes and Genomes (KEGG). Principal component analysis (PCA), enrichment analysis for the differential metabolites, and construction of random forest models were performed on the online platform MetaboAnalyst 4.0 (31).

### Correlation Analysis

Spearman’s correlation coefficients were applied to explore the relationship between differential genera and metabolites, differential metabolites, and phenotypes in R (package “psych”). Correlations between genera, metabolites, and phenotypes were visualized using Cytoscape v3.2.1 (https://www.metaboanalyst.ca).

### Redundancy Analysis

Redundancy analysis (RDA) was performed to summarize variation in the dataset of differential metabolites that can be explained using the dataset of differential genera within six groups. RDA was performed and visualized in R (package “rda” and “ggplot”). ANOVA was performed to test the significance of each bacteria genus.

### Statistical Analysis

Data are presented as mean ± SD. Statistical analysis between two groups was performed using Student’s *t-*test. The abundances of bacteria and metabolites among the six groups were compared using the Kruskal–Wallis H test. Differences in colonic morphology, mucus barrier, SCFAs, and serum parameters were tested by two-way ANOVA. The *p-*value was adjusted for false discovery rate (FDR) using the Benjamini–Hochberg Procedure, and *p* < 0.05 was considered to indicate statistical significance. Analysis of the datasets was completed with R software (version 3.5.1).

## Results

### Microbial Community in the Gut of Mice After Fecal Microbiota Transplantation

On day 14, decreased alpha diversity (*p* < 0.05) together with the differential patterns of bacterial communities was observed in the gut of mice after treatment with the antibiotic (**Figure S1**). The diversity and composition of fecal microbiota from the donors were assessed (**Figure S2**). No significant difference in alpha diversity was observed, although there was a significant difference in the Adonis index for beta diversity between wild and domestic pig fecal microbiota. LEfSe analysis revealed several significantly enriched genera in wild pig microbiota, including *Lactobacillus*, *Clostridium sensu stricto*, *Clostridium* XIVa, *Clostridium* XI, *Turicibacter*, *Escherichia/Shigella*, *Corynebacterium*, *Slackia*, and *Cellulosilyticum*. Domestic pig microbiota, on the other hand, had significantly higher levels of *Prevotella*, *Barnesiella*, *Faecalibacterium*, and *Gemmiger*.

Microbial communities in the gut were assessed after the FMT treatment of mice on day 28. The Shannon index was significantly lower (*p* < 0.01) in the gut of the mice after FMT than that in the CON group. Mice in the WM group also had a significantly lower Shannon index than that in the DM group (*p* < 0.01, **Figure 1C**). Clear separation was observed between the WM group and the CON group (PCoA1 = 66.41%, *p* < 0.01, **Figure 1D**) when weighted UniFrac was used to evaluate the beta diversity of bacterial communities in the gut. Bacteria with top 10 and 20 relative abundances at the phylum (**Figure 1E**) and genus (**Figure 1F**) levels, respectively, are shown. *Bacteroidetes* accounts for the highest relative abundance in both the CON and DM groups, while *Verrucomicrobia* shows the highest relative abundance in the WM group. At the genus level, the WM group had a significantly higher relative abundance of *Akkermansia* than the CON and DM groups, whereas the DM group was significantly higher than the CON group (**Figure S3**).

### Profiling of Fecal Short-Chain Fatty Acids After Dietary Intervention

**Figure 2:**
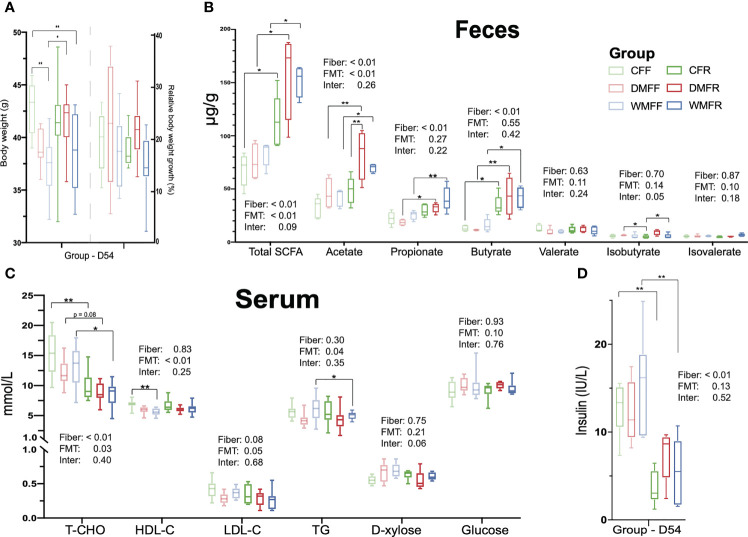
Effect of fecal microbiota transplantation (FMT) together with dietary fiber intervention on mice at day 54. **(A)** Box plots of body weight. **(B)** Fecal short-chain fatty acids (SCFAs). **(C, D)** Serum biochemical characteristics in mice. Statistical differences among individual groups were evaluated using one-way ANOVA, and significance is defined as *p < 0.05; **p < 0.01.

Body weight together with fecal SCFAs was determined after dietary intervention on day 54. The CFF group showed the highest body weight within the six groups and was significantly higher (*p* < 0.05) than the WMFF and WMFR groups (**Figure 2A**). In terms of SCFA levels in the feces (**Figure 2B**), the DMFR and WMFR groups had a significantly higher (*p* < 0.05) content of SCFAs, acetate, propionate, and butyrate in the feces than the DMFF and WMFF groups, respectively. The CFR group had significantly higher (*p* < 0.05) total SCFAs and butyrate in the feces than the CFF group. Moreover, the DMFR group had significantly higher (*p* < 0.05) acetate in the feces than the CFR group.

### Characterization of Serum Parameters After Diet Intervention

Serum parameters were measured within the six groups after dietary intervention on day 54. As shown in **Figure 2C**, serum TC concentration was significantly lower (*p* < 0.05) in the CFR and WMFR groups than in the CFF and WMFF groups and trending lower in the DMFR group than the DMFF group (*p* = 0.08). The WMFF group had significantly lower (*p* < 0.05) serum HDL-C than the CFF group. Serum TG was significantly lower (*p* < 0.05) in the WMFR group than in the WMFF group. There was no significant difference in serum HDL-C, LDL-C, d-xylose, and glucose levels between the CFF and CFR, DMFF and DMFR, or WMFF and WMFR groups, respectively. Serum insulin level was significantly lower (*p* < 0.05) in the CFR and WMFR groups than in the CFF and WMFF groups, respectively (**Figure 2D**).

### Colonic Morphology and the Mucus Barrier

**Figure 3:**
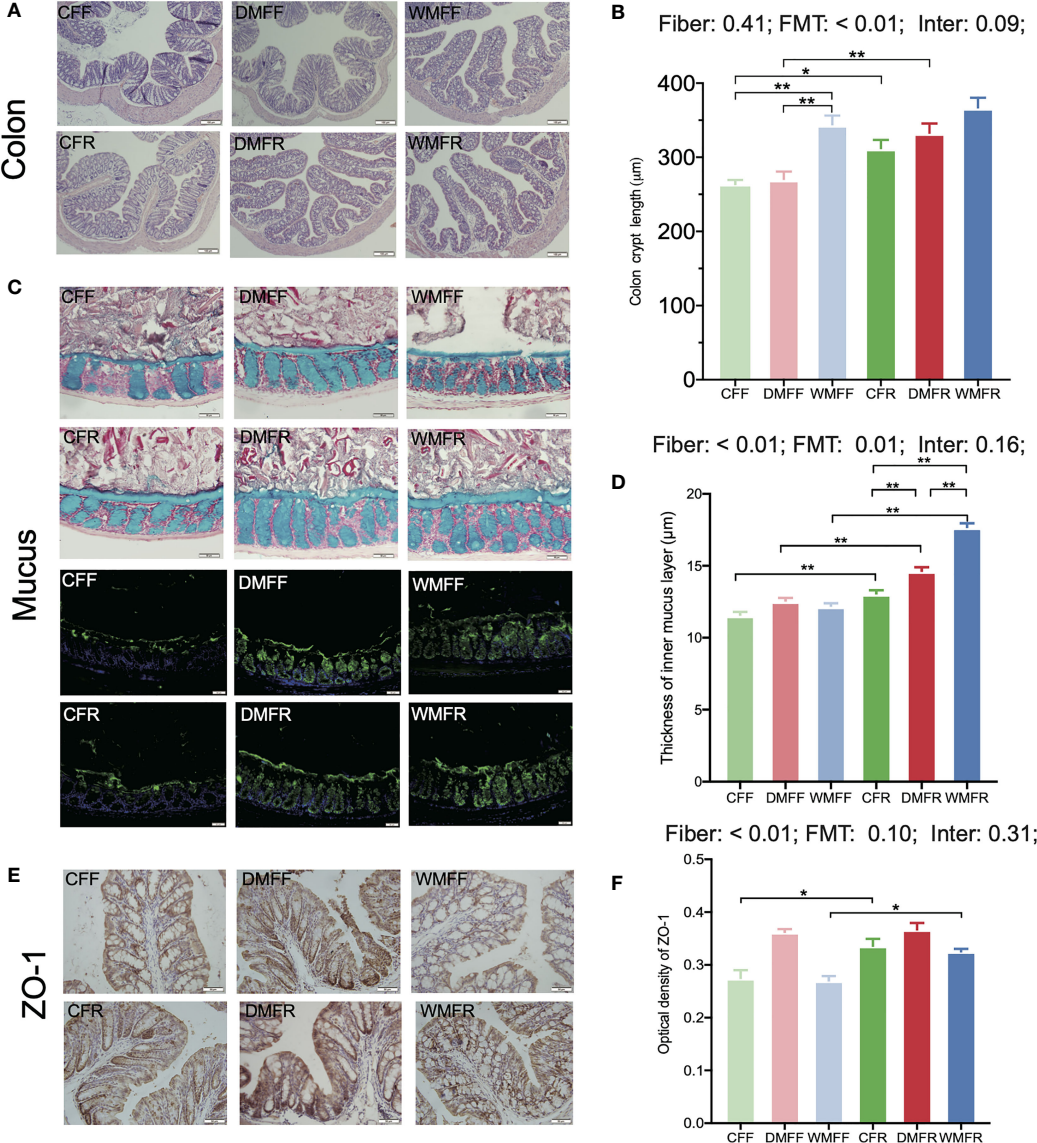
Gut barrier in the mice fed a fiber-free (CFF) or fiber-rich (CFR) diet, or fecal microbiota transplantation (FMT) from domestic pigs and fed with a fiber-free (DMFF) or fiber-rich (DMFR) diet, or FMT from wild pigs and fed with a fiber-free (WMFF) or fiber-rich (WMFR) diet. **(A, B)** Gut morphology, **(C, D)** mucin barrier, and **(E, F)** tight junction protein in the colon of mice. Statistical differences among individual groups were evaluated using one-way ANOVA, and significance is defined as *p < 0.05; **p < 0.01.

The H&E section showed that mice with an FR diet had better colonic morphology (**Figure 3A**). Colonic crypt length was significantly higher (*p* < 0.05) in the CFR group than the CFF group, and in the DMFR group than the DMFF group (**Figure 3B**). Among the groups with the FF diet, the WMFF group had a significantly higher colonic crypt length than the CFF and DMFF groups (**Figure 3B**).

The mucus layer thickness was measured in Alcian blue-stained sections and by immunofluorescence staining for MUC2 (**Figure 3C**). The analysis revealed that mucus thickness was significantly higher (*p* < 0.05) with an FR diet than in the FF groups (**Figure 3D**). Within the FR groups, colonic mucus thickness was significantly higher (*p* < 0.05) in the DMFR and WMFR groups than in the CFR group. The WMFR group also had significantly higher (*p* < 0.05) colonic mucus thickness than the DMFR group.

The immunohistochemistry analysis showed that ZO-1 expression was significantly higher (*p* < 0.05) in the CFR and WMFR groups than in the CFF and WMFF groups, respectively, but did not show any significant difference between the DMFF and DMFR groups (**Figures 3E, F**). There was no significant difference in Claudin-3 and Occludin expression among different treatments (**Figure S4**).

### Microbial Community of Mice Following Fecal Microbiota Transplantation and Dietary Intervention

**Figure 4:**
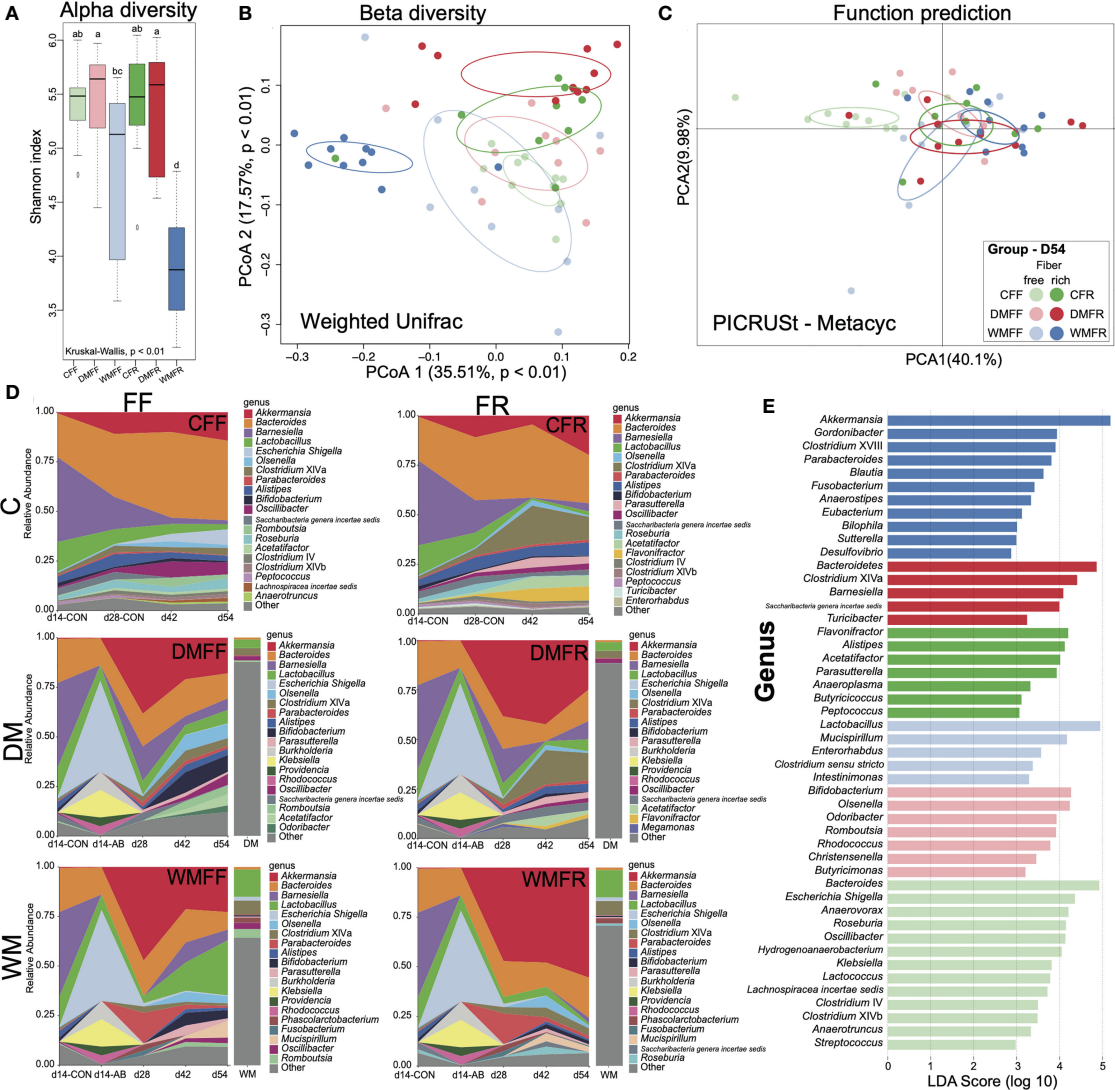
Bacteria communities in mice treated with fecal microbiota transplantation (FMT) together with dietary fiber intervention at day 54. **(A)** Boxplot of Shannon index of gut microbiota in mice. **(B)** Principal coordinates analysis (PCoA) of gut microbiota structures in the mice between six groups. **(C)** Principal component analysis (PCA) of the functional content of gut microbiota predicted by PICRUSt in the mice among six groups. **(D)** Changes in the relative abundance of gut microbiota over time in mice oscillated among FMT and fiber feeding. **(E)** Bar charts showed the linear discriminant analysis (LDA) score (>2) for the gut microbiota in the mice. Kruskal–Wallis test with an adjusted *p-*value of <0.01 was used. ^a,b,c,d^ Means within the same row followed by different superscripts differ at *p* < 0.01.

The composition and diversity of fecal microbiota within the six groups were assessed on day 54 at the end of the study. Alpha diversity is compared and shown in **Figure 4A**. FMT-treated mice with donors from domestic pigs (DMFF and DMFR) showed significantly higher (*p* < 0.05) Shannon index of gut microbiota than mice with donors from wild pigs (WMFF and WMFR), while the WMFR group had significantly lower (*p* < 0.05) Shannon index than the other five groups. Weighted-UniFrac principal coordinates analysis (PCoA) was used for the beta-diversity analysis and revealed that the WMFR group showed clear separation from the other five groups (**Figure 4B**). With PICRUSt applied for the prediction of function in the gut (**Figure 4C**), PCA revealed no separation between FMT-treated mice from different donors.

Fecal microbial community dynamics analysis showed that with antibiotic treatment, FMT, and dietary intervention, several bacterial taxa rapidly and reproducibly changed in relative abundance (**Figure 4D**). Four genera—*Akkermansia*, *Bacteroides*, *Barnesiella*, and *Escherichia*—were highly responsive to microbiota colonization and change of diet. Mice with FMT (DM or WM) had a higher relative abundance of *Akkermansia* but a lower relative abundance of *Bacteroides* than the two control groups. *Akkermansia* abundance in the WMFR group was higher than in the WMFF group at all points in time. The relative abundance of *Clostridium* cluster XIVa increased in mice fed the FR diet (CFR and DMFR), whereas the relative abundance of *Escherichia* increased in mice fed the FF diet. *Bifidobacterium* increased in the DMFF and WMFF groups at the later time points (days 42 and 54). *Mucispirillum* increased from day 42 to day 54 in the WMFF and WMFR groups.

Gut microbiota were dominated by the *Bacteroides* and *Escherichia/Shigella* in the CFF group while dominated by *Bacteroides*, *Flavonifractor*, *Alistipes*, and *Acetatifactor* in the CFR group (**Figure 4D**). In other groups, the DMFF group was dominated by *Bifidobacterium* and *Olsenella*, and the DMFR group was dominated by *Clostridium* cluster XIVa, *Barnesiella*, and Saccharibacteria. The WMFF group was dominated by *Lactobacillus* and *Mucispirillum*, and the WMFR group was dominated by *Akkermansia*.

LEfSe analysis shows differential bacteria genera within the six groups in **Figure 4E**. In the WMFR group, the genus *Akkermansia* with an LDA score over 5 had a significantly higher (*p* < 0.05) relative abundance than the other five groups (**Figure S5**). Within the WMFF group, *Lactobacillus* showed the highest LDA score together with a significantly higher relative abundance than the other five groups (**Figure S5**). *Bacteroidetes* and *Bifidobacterium* showed the highest LDA score in the DMFR and DMFF groups, respectively. The highest LDA scores of *Flavonifractor* and *Bacteroides* were shown in the gut of the CFR and CFF groups, respectively. The CFR group had a significantly higher (*p* < 0.05) relative abundance of genus *Alistipes* than the other five groups.

### Metabolism Profiles of Feces in Mice Treated With Fecal Microbiota Transplantation and Diet Intervention

**Figure 5:**
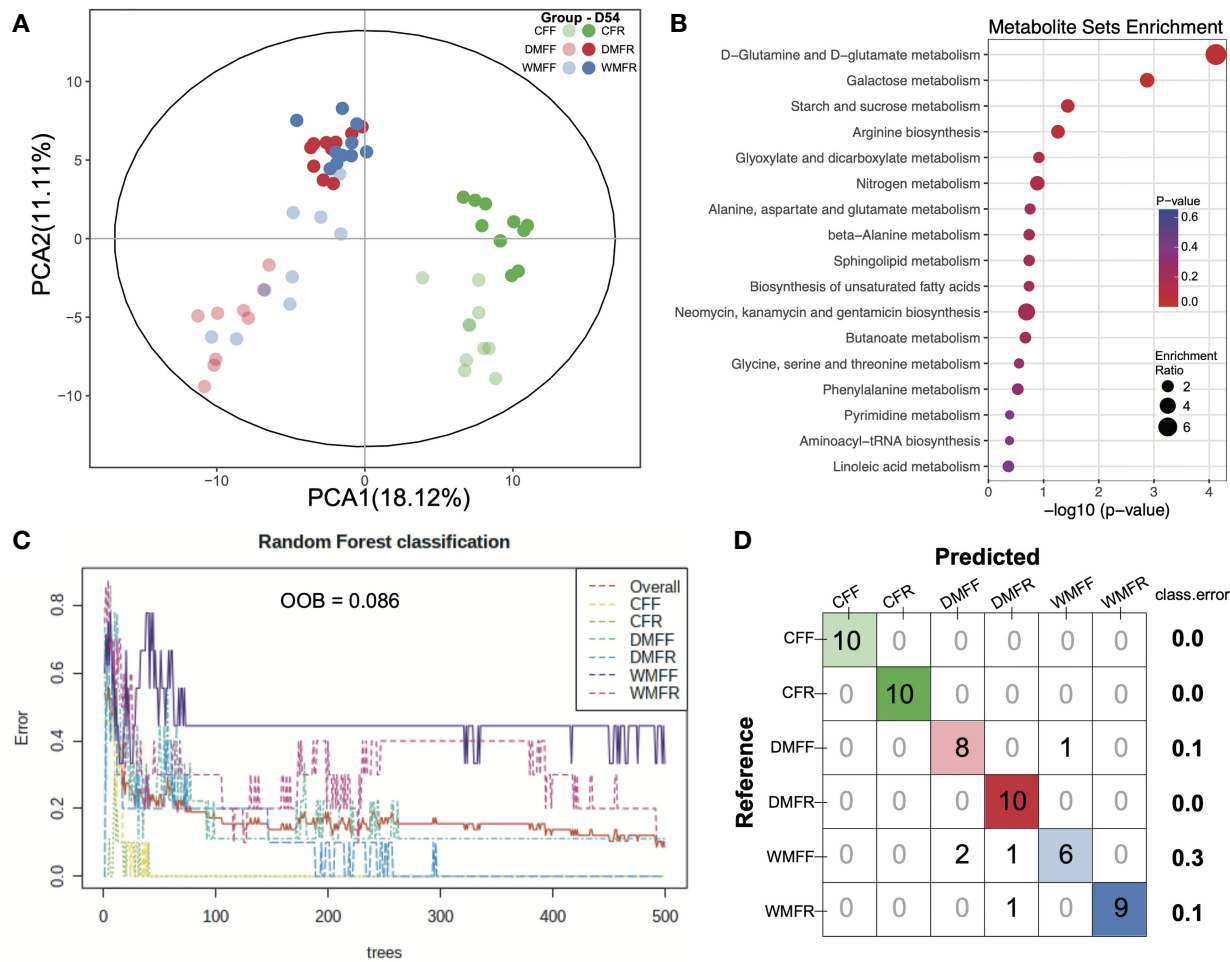
Metabolism in the gut in mice treated with fecal microbiota transplantation (FMT) together with dietary fiber intervention at day 54. **(A)** Principal component analysis (PCA) revealed the profiles of metabolites in the gut of mice within the six groups. **(B)** Enrichment pathways for the differential metabolites in the gut. **(C, D)** Random forest model was constructed, and a confusion matrix was applied with the dataset of metabolites in the gut of mice.

Untargeted metabolomics was performed to evaluate whether shifts in the microbiota were mirrored by changes in the gut metabolite landscape. Based on the LECO/Fiehn Metabolomics Library, a total of 521 metabolite peaks were identified across the six groups, of which 250 were known (**Table S2**). The PCA of GC-TOF/MS metabolic profiles of feces showed that CFF and CFR formed a cluster, while another cluster was observed with regard to DMFR and WMFR (**Figure 5A**). Differential metabolites within the six groups (**Table S3**) were used for the enrichment analysis, and pathways with a *p-*value <0.01 are shown in **Figure 5B**. A total of two pathways were observed with a *p-*value <0.01, namely, d-glutamine and d-glutamate metabolism, and galactose metabolism. The random forest model was also constructed with the dataset of metabolites in the six groups, and the classification results together with the confusion matrix are shown in **Figures 5C, D**. The out of bag (OOB) value was 0.086 and 194 metabolites (**Table S4**) with mean decreased accuracy (MDA) over 0 were observed in the model. Classification errors were 0 in the classification for the CFF, CFR, and DMFR groups and 0.3 of the error that occurred in the classification of mice in the WMFF group.

### Correlation of the Phenotype, Metabolite, and Bacteria of Mice

**Figure 6:**
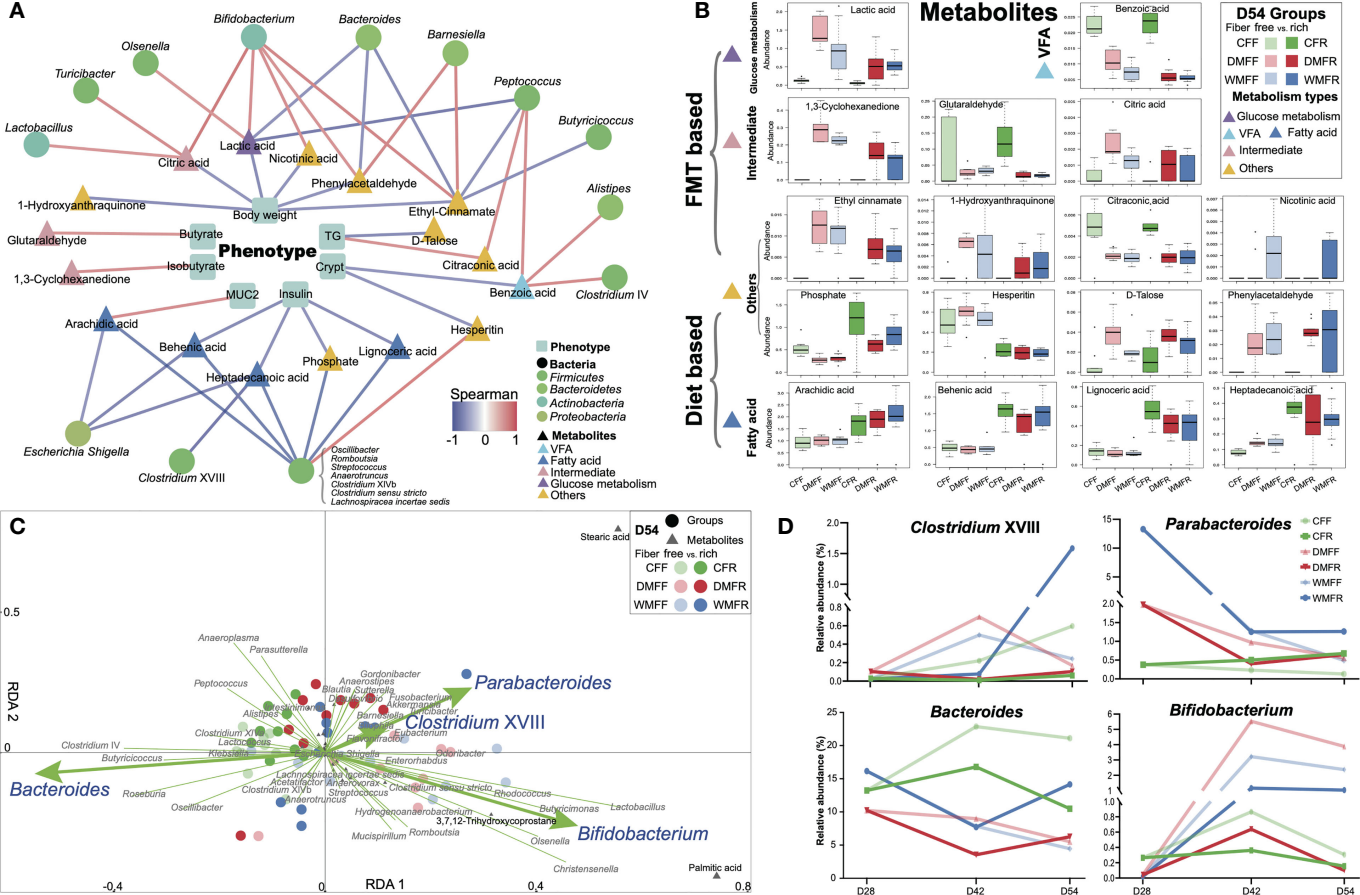
Differential bacteria and metabolites in the gut contribute to the phenotypes of mice treated with fecal microbiota transplantation (FMT) together with dietary fiber intervention at day 54. **(A)** Correlation between differential metabolites and phenotypes (*p* < 0.05), differential bacteria, and metabolites in the gut of mice (*p* < 0.01). **(B)** Box plot of the differential metabolites correlated with gut microbiota and phenotypes of mice. **(C)** Redundancy analysis (RDA) revealed that gut microbiota contributes to variation of the metabolites in the gut of mice. **(D)** Alteration in the relative abundance of the differential gut microbiota in RDA within the dietary fiber intervention after FMT of the mice.

**Figure 7:**
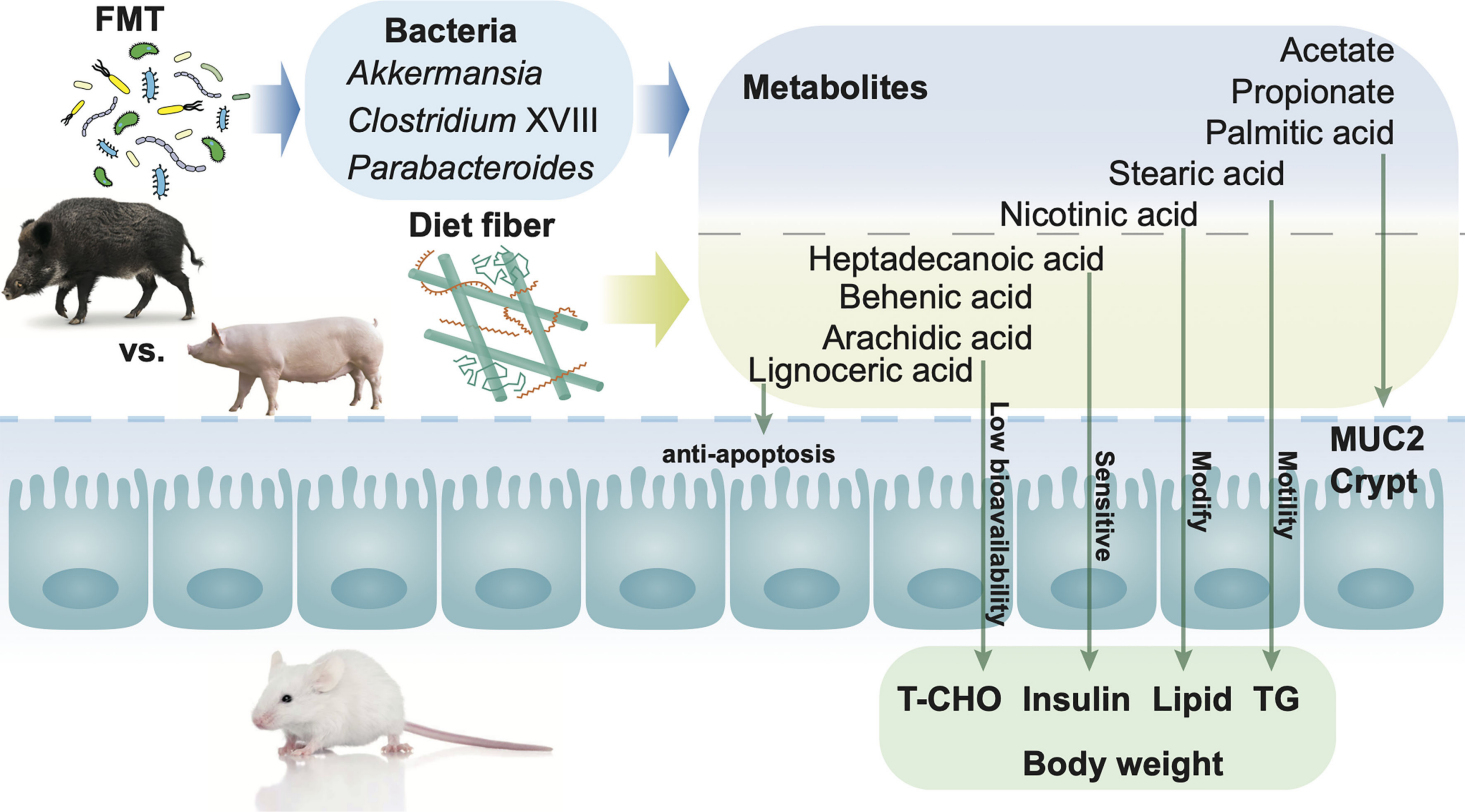
Mechanisms of fecal microbiota transplantation (FMT) together with dietary fiber intervention contribute to the alteration of metabolites in the gut, leading to modification of gut barrier function and serum characteristics in mice. Both FMT and dietary fiber intervention contributed to the alteration of the host health, *via* metabolites in the gut. Dietary intervention with high fiber led to the alteration of metabolites such as lignoceric acid, behenic acid, arachidic acid, and heptadecanoic acid in the gut. Mice FMT from wild pigs together with a high fiber diet showed a higher abundance of *Akkermansia*, *Parabacteroides*, and *Clostridium* XVIII in the gut. The alteration of metabolites together with bacteria in the gut plays an important role in the improvement of mucus barrier function as well as the decrease in body weight, total cholesterol (TC), and insulin.

Spearman’s correlation was performed between phenotypes and metabolites (*p* < 0.05), as well as metabolites and genera (*p* < 0.01), and shown in **Figure 6A**. The body weight, crypt length in the gut, and insulin in the serum of mice all showed a negative correlation with metabolites in the gut. The concentration of butyrate and isobutyrate as well as MUC2 in the gut showed a positive correlation with the glutaraldehyde, 1,3-cyclohexanedione, and arachidic acid, respectively. Within the bacteria genera correlated with metabolites in the gut, most of the genera were from *Firmicutes*. Moreover, *Bifidobacterium* and *Lactobacillus* from Actinobacteria and *Escherichia/Shigella* from Proteobacteria were positively and negatively correlated with metabolites, respectively. The abundance of differential metabolites within groups is shown in **Figure 6B**. Metabolites belonging to SCFA, glucose metabolism, and intermediate showed “FMT-based” changing patterns, while metabolites belonging to fatty acid showed “diet-based” changing patterns. RDA revealed that bacteria genera *Parabacteroides*, *Clostridium* XVIII, *Bifidobacterium*, and *Bacteroides* showed significant (*p* < 0.05) explanatory variables for the variation in metabolites within the six groups (**Figure 6C**). The alteration of relative abundance of these bacteria genera during the dietary intervention within the six groups is shown in **Figure 6D**. Metabolites such as lignoceric acid, behenic acid, arachidic acid, and heptadecanoic acid can be affected by dietary fiber, while acetate, propionate, palmitic acid, stearic acid, and nicotinic acid can be affected by FMT, including *Akkermansia*, *Clostridium* XVIII, and *Parabacteroides* (**Figure 7**). Together, these results suggest that both FMT and dietary intervention can contribute to the alteration of metabolites and thus facilitate host health.

## Discussion

Dietary fiber could shape gut microbiota and further contribute to gut health by means of alteration of metabolites. FMT was confirmed to be an effective practice in shaping the gut microbiota of the recipient and was largely influenced by the selection of donors (32). The response of gut microbiota from different donors to dietary fiber in recipients remains a subject for further investigation. Hence, in our study, FMT was implemented, and the fecal microbiota of domestic and wild pigs were transplanted into mice for the establishment of the models for recipients of FMT from different donors. Domestic pigs (DLY) and wild pigs (W) were selected as donors for FMT, and we observed a clear separation of the gut microbiota communities between DLY and W. Similar to previous studies that reported on the gut microbiota of W and DLY (33, 34), there were distinct differences between the gut microbiota of DLY and W due to the different living environments and food resources (35) despite that they are the most closely related species to each other. Alteration of gut microbiota was also observed in DM and WM mouse FMT from DLY and W pigs, respectively, which suggests the influence of donors on the recipient.

After FMT, a diet with different soluble (inulin) and insoluble (cellulose) fiber contents was introduced in the CON, DM, and WM mice for 4 weeks. Dietary fiber, especially soluble fiber, can be fermented by gut microbiota, and SCFAs, including acetate, propionate, and butyrate, are major products (36) in mice (37), pigs (38), and adults (39). Lower concentrations of insulin levels in serum were observed when humans supplemented their diets with inulin or cellulose (40), and a cholesterol-lowering effect of dietary fiber was revealed in a meta-analysis of 67 trials (41). Similar results were observed in our study when mice received a higher content of dietary fiber regardless of the FMT treatment. Reduced weight gain and improvement in glucose homeostasis were observed when mice were administrated acetate (42), propionate (43), or butyrate (44), respectively.

The combination of a single-dose FMT and daily low-fermentable fiber intervention for 6 weeks improved insulin sensitivity in patients suffering from severe obesity and metabolic syndrome (45). In our study, mice that received higher amounts of dietary fiber did not show a differential body weight gain despite the lower concentration of T-CHO and insulin in the serum, which could be the reason that dietary fiber often has a long-term effect on weight gain (46), whereas the intervention of dietary fiber only lasted for 4 weeks. In addition, the effect of FMT was observed in the gut morphology of mice. The gut barrier is responsible for both nutrient absorption and for defending the gut from dangerous macromolecules, thus playing a critical role in host health (47). Stem cells located at the bottom of crypts could maintain the self-renewal of the gut epithelium (48), which protects against potential pathogens and toxins (49). However, the growth of stem cells could be inhibited by butyrate; thus, the architecture, especially crypt length, protects stem cells from the effect of butyrate (50). CFR and DMFR mice treated with higher amounts of dietary fiber showed higher crypt length than CFF and DMFF, while WMFF showed higher crypt length than CFF and DMFF, indicating the effect of FMT on the gut barrier. As one of the first lines of protection in the gut barrier, the mucus layer guards against external attacks by creating a coat that covers the epithelium to maintain gut homeostasis (51–53). Higher dietary fiber supplementation usually contributes to the mucus barrier, partly due to the rise of SCFAs, especially acetate and butyrate, which stimulate MUC2 expression and increase the production and secretion of mucus (54, 55). In this study, a differential thickness of mucus layer was not only observed between mice with different dietary fiber contents but also in mice FMT from different donors, suggesting the vital role of gut microbiota in gut health (1, 56). However, a lower density of ZO-1 was observed in WMFR, which was not consistent with the highest colon crypt length and mucus thickness and warrant further investigation.

The gut microbiota communities together with their metabolites in the gut of mice were investigated and revealed a combination effect of FMT and dietary fiber. Donor selection can be key in the FMT process, which in the end influences the effectiveness of FMT (32), while proper diet intervention could contribute long-term and beneficial effects after FMT (57, 58). The gut microbiota of WMFR mice showed a differential pattern compared with the other five groups, suggesting the positive effect of the combination of FMT and dietary fiber. Enrichment of the genus *Akkermansia* was observed in the WM group on day 28 and the WMFR group on day 54 and has been shown to be a promising probiotic in both rodents and humans (59–61). An *in vitro* study revealed that *Akkermansia* could improve gut health by adhering to the intestinal epithelium and strengthening enterocyte monolayer integrity (62). Moreover, *Parabacteroides* were also observed in the WM group on day 28 and the WMFR group on day 54. Both *in vitro* and *in vivo* studies revealed *Parabacteroides* as a probiotic that could reinforce epithelium and modulate host metabolism in the gut (63, 64). Oral administration of *Parabacteroides* coupled with prebiotics could enhance intestinal integrity and improve insulin sensitivity in mice receiving a high-fat diet (65). FMT from wild pigs with a higher abundance of *Akkermansia* and *Parabacteroides* coupled with dietary fiber intervention could consistently nourish these kinds of bacteria and thus contribute to mucus layer thickness as well as the gut barrier in mice.

Although previous studies revealed the importance of SCFAs in crypt depth (66), mucus layer (67), and tight junction (68) in the gut, SCFAs showed no difference between mice FMT from domestic pigs or wild pigs when higher dietary fiber content was introduced, suggesting that other microbiota-derived metabolites also contribute to the gut barrier. Unique patterns of gut metabolites were observed among the six groups, and several metabolites have been observed to be correlated with FMT, including palmitic acid, stearic acid, and nicotinic acid. An *in vivo* study revealed that palmitic acid decreases MUC2 production mainly by alleviating the endoplasmic reticulum stress in goblet cells (69). In accordance with our study, a higher abundance of palmitic acid together with the lower thickness of MUC2 was observed in both the WMFF and DMFF groups. Stearic acid, which contributes to motility and improves gut health (70), was observed to be enriched in the WMFR and DMFR groups. Mice with FMT from wild pigs showed a higher abundance of nicotinic acid, which has been reported to be crucial in the inflammatory of the gut (71).

Apart from the metabolites altered by FMT, abundances of fatty acids such as lignoceric acid, behenic acid, arachidic acid, and heptadecanoic acid, were observed to be affected by the dietary fiber intervention. Lignoceric acid has been reported to have a function such as anti-inflammatory effects possibility *via* α-oxidation (72). Behenic acid and arachidic acid have low bioavailability, which usually leads to a reduction in absorption and as a result to the reduction in T-CHO (73). We acknowledge that the antibiotic treatment could influence the effects of both FMT and dietary intervention in some of the results observed, especially the lower alpha diversity in FMT mice, although minor effects of antibiotic pretreatment in FMT were reported (74).

Collectively, dynamic interactions existed between FMT and dietary fiber intervention in the mouse model, suggesting that donor selection combined with dietary fiber intervention may facilitate the benefits of FMT on the host. Our results suggest that specific bacteria from donors along with high dietary fiber supplementation altered metabolites and, consequently, contributed to gut health. This study highlights the importance of beneficial bacteria received by individuals through a high dietary fiber diet, which provides a novel insight into the diet manipulation in both donors and recipients in FMT.

## Data Availability Statement

The datasets presented in this study can be found in online repositories. The names of the repository/repositories and accession number(s) can be found in the article/**Supplementary Material**.

## Ethics Statement

The animal study was reviewed and approved by the Animal Care and Use Committee of Zhejiang University.

## Author Contributions

HW designed the experiments. JC, YFZ, and YM performed the experiments. HW, YZ, JC, and JL analyzed the data. HW and YZ wrote and revised the main manuscript. All authors read and approved the final manuscript.

## Funding

This study was supported by grants from the National Natural Science Foundation of China (31672430), the Natural Science Foundation of Zhejiang Province (Z19C170001), the National Key Research and Development Program of China (2017YFD0500502), and the Funds of Ten Thousand People Plan.

## Conflict of Interest

The authors declare that the research was conducted in the absence of any commercial or financial relationships that could be construed as a potential conflict of interest.

## Publisher’s Note

All claims expressed in this article are solely those of the authors and do not necessarily represent those of their affiliated organizations, or those of the publisher, the editors and the reviewers. Any product that may be evaluated in this article, or claim that may be made by its manufacturer, is not guaranteed or endorsed by the publisher.

## Supplementary Material

Supplementary Figure 1 Alpha diversity of Shannon **(A)** and Chao 1 **(B)** index, beta diversity **(C)**, OTU composition **(D)**, unique and shared **(E)** and differential **(F)** of bacteria in the gut of mice between the control (CON) and antibiotics (AB) group.

Supplementary Figure 2 Alpha diversity of Shannon **(A)** and Chao 1 **(B)** index, beta diversity **(C)**, composition **(D)**, cladogram **(E)** and LDA score **(F)** of bacteria LEfSe analysis and relative abundance **(G)** of bacteria in the feces of wild (W) and domestic (DLY) pigs.

Supplementary Figure 3 Fecal bacteria at genus level in the control (CON) group and fecal microbiota transplantation (FMT) from domestic pigs (DM) and wild pigs (WM) at day 28. **(A)** Relative abundance of top 20 bacteria within CON, DM, and WM mice. **(B)** LDA score of fecal bacteria genera in CON, DM, and WM mice (LDA score > 2).

Supplementary Figure 4 Immunochemistry of tight junction protein of **(A)** Claudin-3 and **(B)** Occludin in the colon of mice within six groups.

Supplementary Figure 5 Differential fecal bacteria of mice within six groups at day 54.
