# Supplementary figures and images for: Fecal Microbiota Transplantation Donor and Dietary Fiber Intervention Collectively Contribute to Gut Health in a Mouse Model
Source: Front Immunol. 2022 Feb 3;13:842669. doi: 10.3389/fimmu.2022.842669 (PMC8852624; doi:10.3389/fimmu.2022.842669)

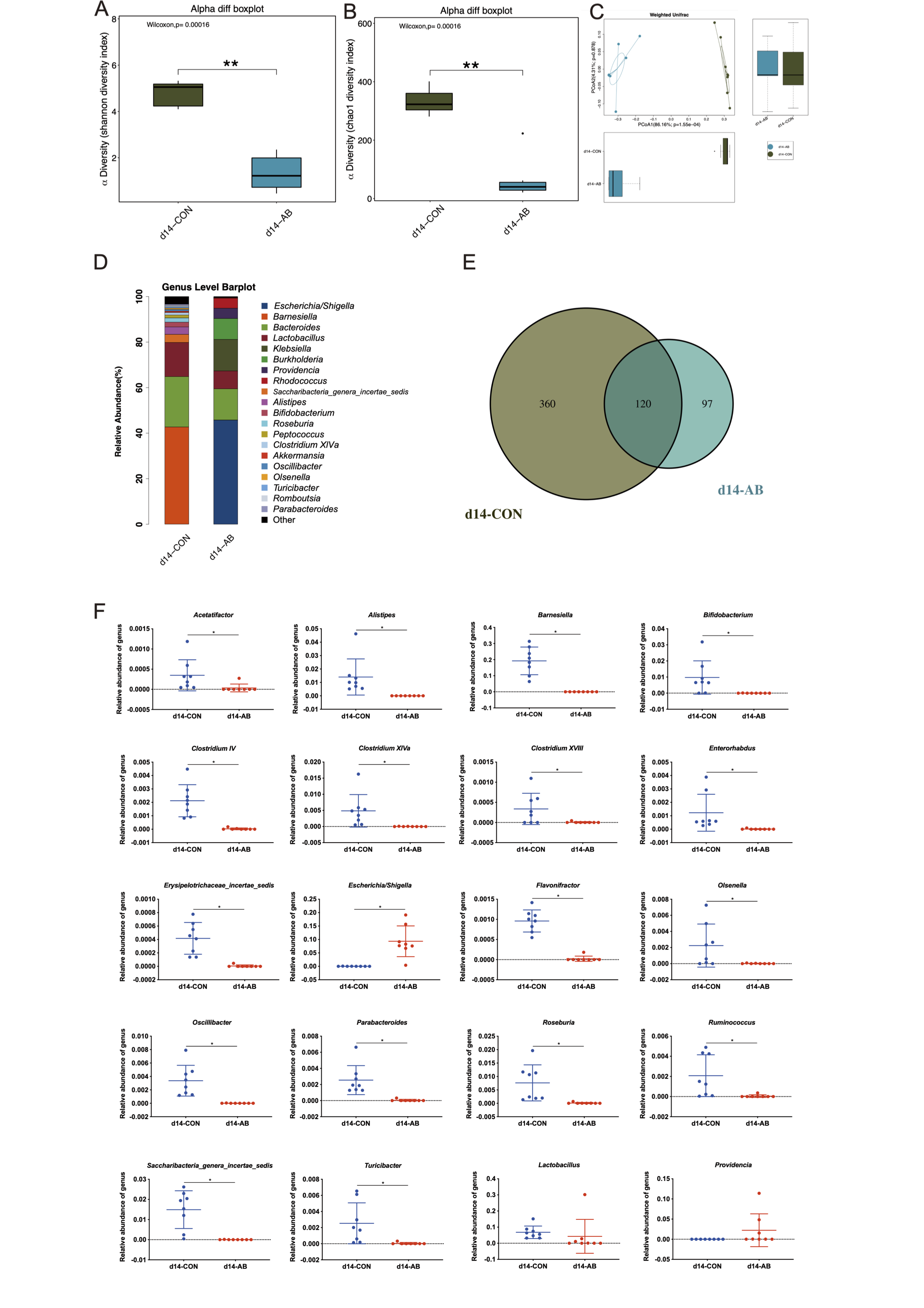

Supplement: Supplementary Figure 1 — Alpha diversity of Shannon (A) and Chao 1 (B) index, beta diversity (C), OTU composition (D), unique and shared (E) and differential (F) of bacteria in the gut of mice between the control (CON) and antibiotics (AB) group. [file Image_1.tiff]

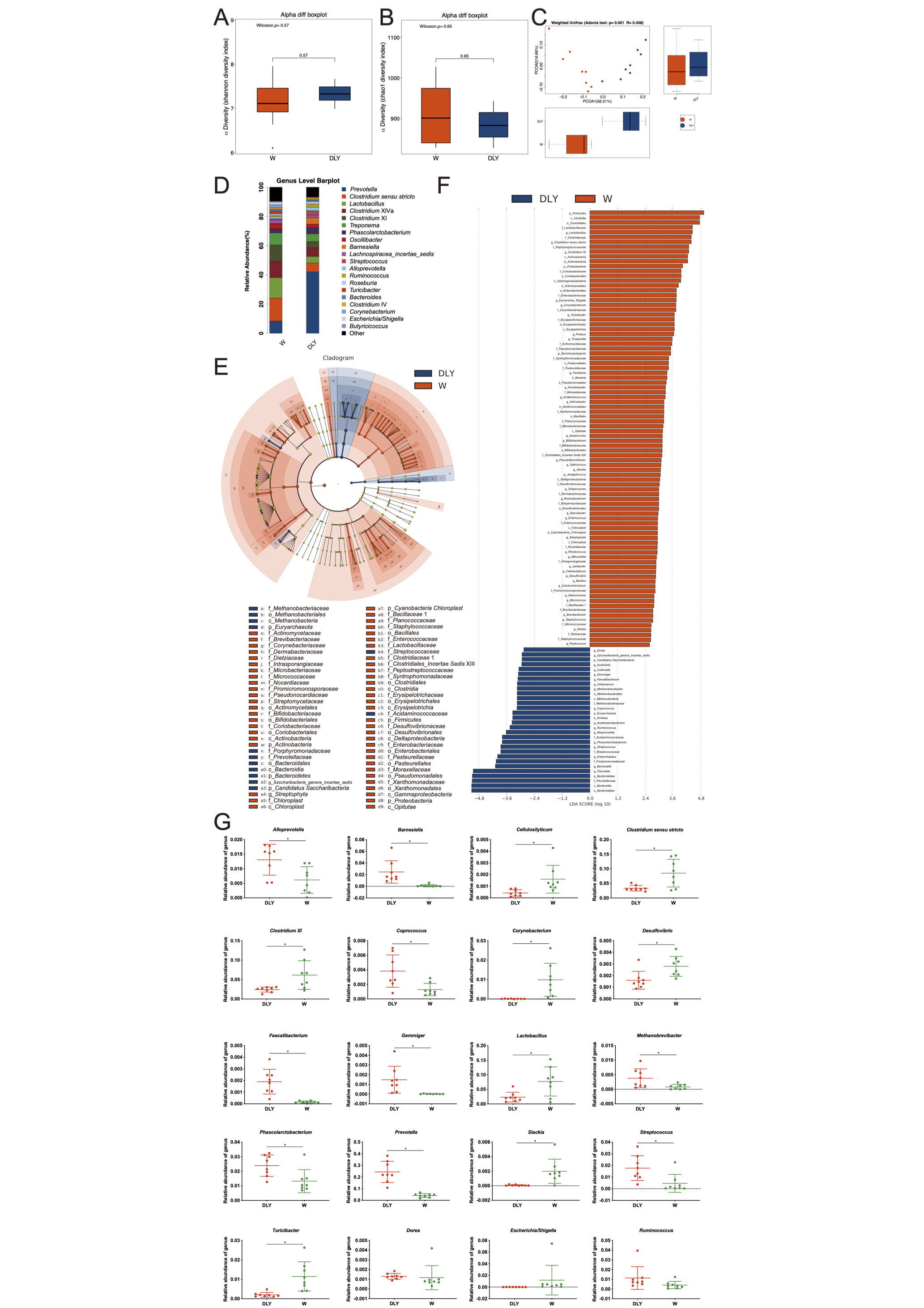

Supplement: Supplementary Figure 2 — Alpha diversity of Shannon (A) and Chao 1 (B) index, beta diversity (C), composition (D), cladogram (E) and LDA score (F) of bacteria LEfSe analysis and relative abundance (G) of bacteria in the feces of wild (W) and domestic (DLY) pigs. [file Image_2.tiff]

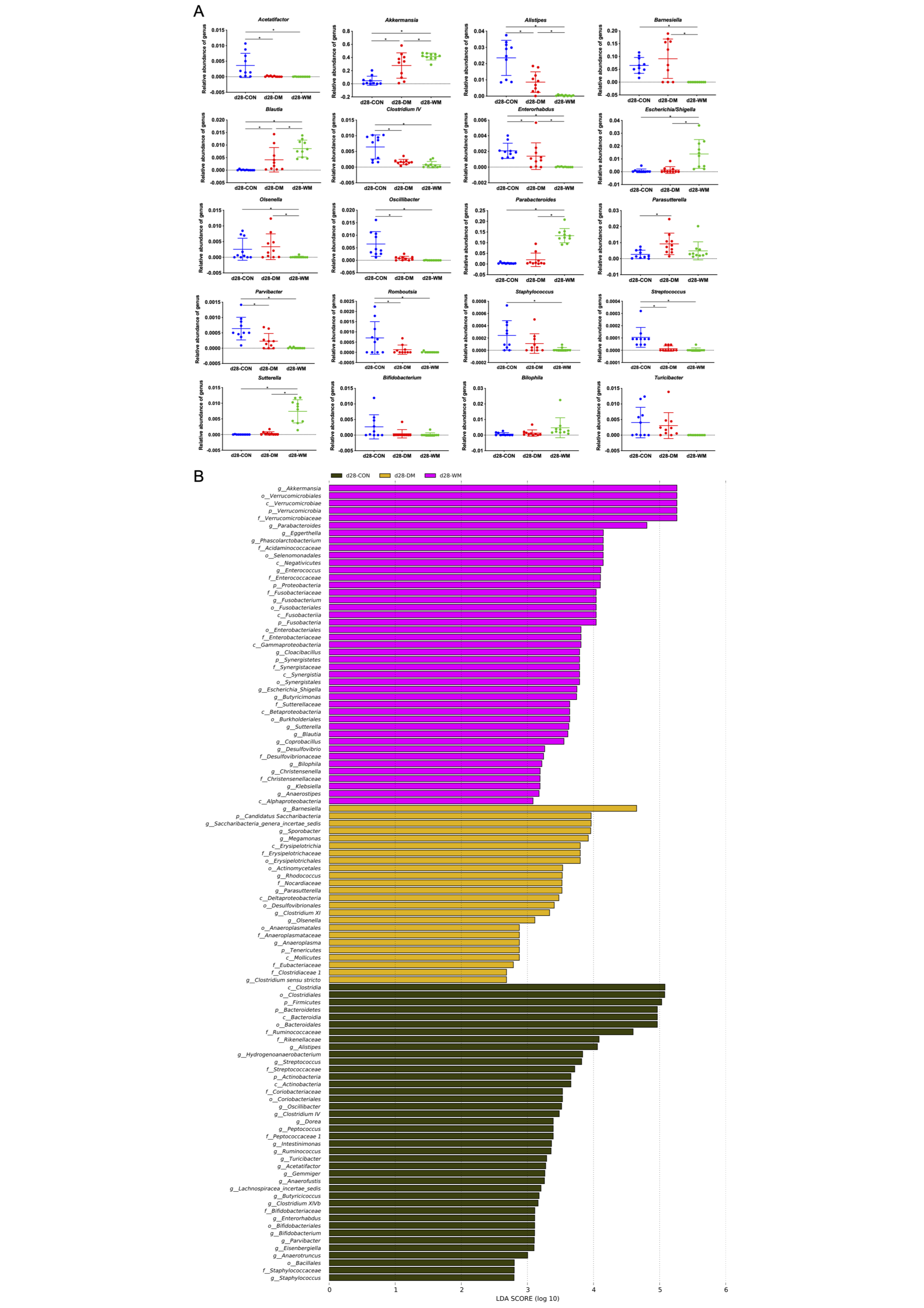

Supplement: Supplementary Figure 3 — Fecal bacteria at genus level in the control (CON) group and fecal microbiota transplantation (FMT) from domestic pigs (DM) and wild pigs (WM) at day 28. (A) Relative abundance of top 20 bacteria within CON, DM, and WM mice. (B) LDA score of fecal bacteria genera in CON, DM, and WM mice (LDA score > 2). [file Image_3.tiff]

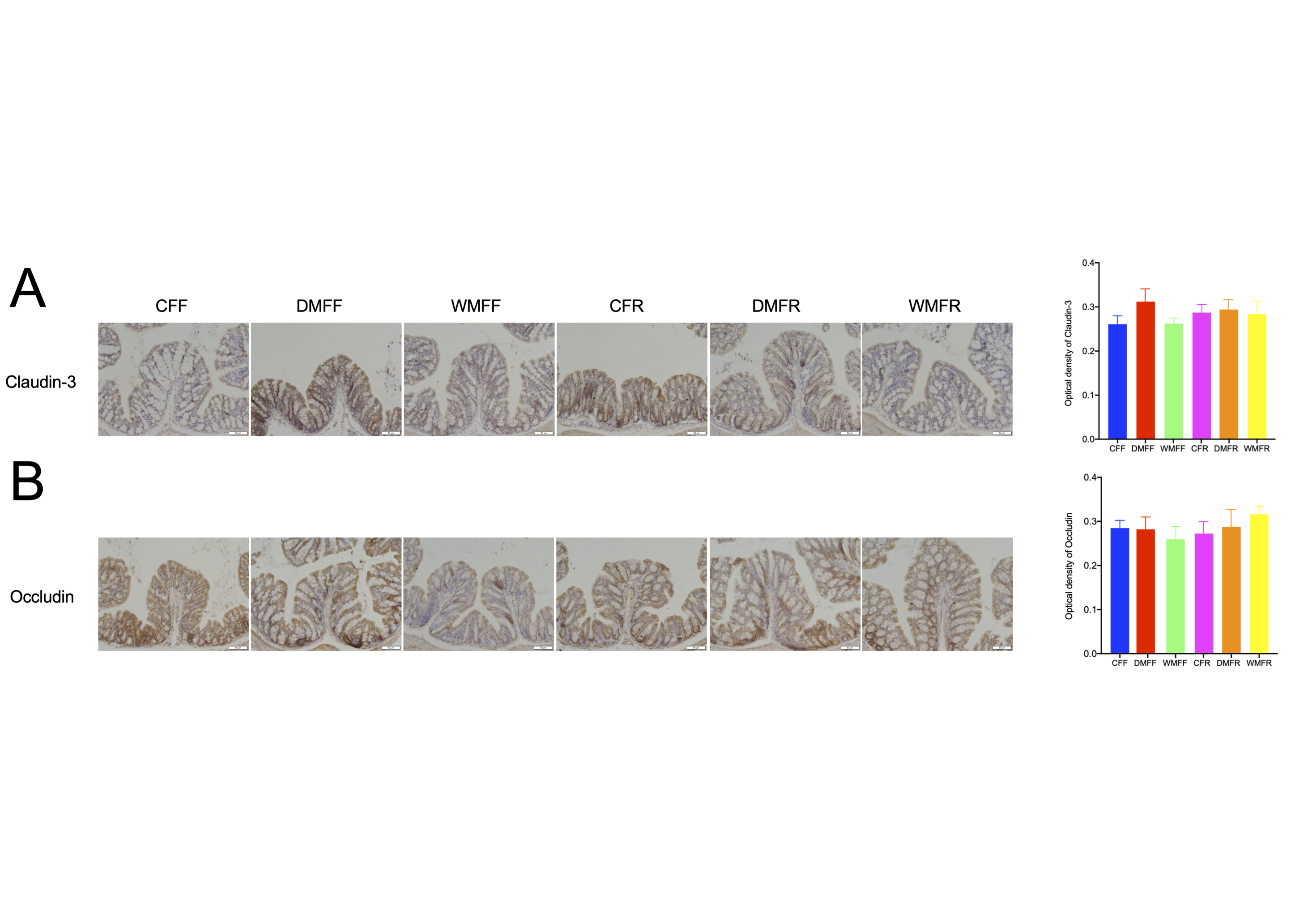

Supplement: Supplementary Figure 4 — Immunochemistry of tight junction protein of (A) Claudin-3 and (B) Occludin in the colon of mice within six groups. [file Image_4.tiff]

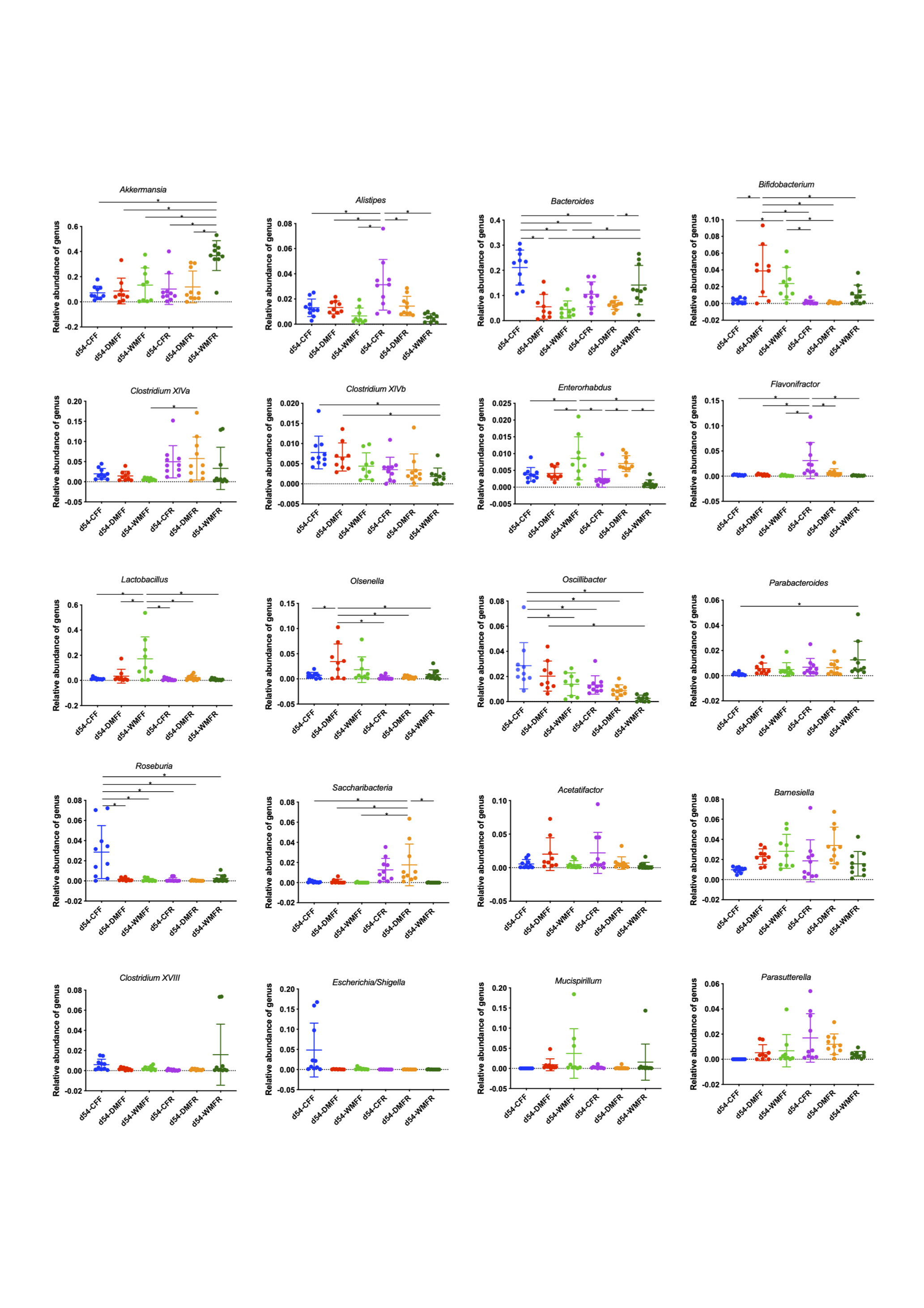

Supplement: Supplementary Figure 5 — Differential fecal bacteria of mice within six groups at day 54. [file Image_5.tiff]
